# Abruptly Autofocusing Vortex Beams for Rapid Controllable Femtosecond Two-Photon Polymerization

**DOI:** 10.3390/ma16134625

**Published:** 2023-06-27

**Authors:** Erse Jia, Chen Xie, Yue Yang, Na Xiao, Minglie Hu

**Affiliations:** 1 Ultrafast Laser Laboratory, Key Laboratory of Opto-Electronic Information Technical Science of Ministry of Education, School of Precision Instruments and Opto-Electronics Engineering, Tianjin University, Tianjin 300072, China; jiaerse@tju.edu.cn (E.J.); yangyue0819@tju.edu.cn (Y.Y.); huminglie@tju.edu.cn (M.H.); 2 Research Center for Biomedical Optics and Molecular Imaging, Shenzhen Key Laboratory for Molecular Imaging, Guangdong Provincial Key Laboratory of Biomedical Optical Imaging Technology, Shenzhen Institute of Advanced Technology, Chinese Academy of Sciences, Shenzhen 518055, China; n.xiao@siat.ac.cn

**Keywords:** femtosecond laser, two-photon polymerization, abruptly autofocusing vortex beams, spatial light modulator

## Abstract

Micro-fabrication based on structured-beam-assisted Two-Photon Polymerization (2 PP) provides a rapid and flexible method for the manufacture of microstructures with complex morphologies. The tunable Abruptly Autofocusing Vortex (AAFV) beams were designed theoretically and generated experimentally based on a single-phase-only Spatial Light Modulator (SLM). Their specific spatial intensity distributions were further utilized to assist the fabrication of a bowl-shaped Three-Dimensional (3D) micro-trap array via 2 PP with a one-step exposure technique. Finally, the fabricated microstructures act as a novel tool for the trapping and spatial positioning of micro-particles with different diameters, which shows potential applications in fiber optics and cell study.

## 1. Introduction

In recent years, the trapping and precise positioning of micro-particles have attracted enormous attention due to their wide applications in microfluidic chip and cell growth kinetics research [1,2,3,4]. The optical tweezer [5,6,7,8,9,10], also known as the single-beam gradient-force optical trap, is a simple and powerful tool to manipulate micro-objects of several microns or even tens of nanometers. However, the generation of optical gradient force often requires a tightly focused laser through a high Numerical Aperture (N.A.) microscope objective, whose high energy density and thermal effects may have adverse effects on living cells and chemical reactions. Therefore, to overcome the aforementioned problems, a variety of microstructures have been designed and manufactured for micro-object manipulation such as hierarchical helical arrays [11], HYAA-chips [12,13], and pre-stressed actuator hinges [14]. The evident drawback is that these manufacture methods require demanding procedures and, most restrictively, lack flexibility and customizability. Therefore, it is still urgent to develop a simple, efficient, and controllable manufacturing method for particle trapping.

Direct laser writing based on Two-Photon Polymerization (2 PP) is a well-established technique based on the high peak power of femtosecond lasers, since the nonlinear two-photon absorption could only be initialized in the focal region of the tightly focused spot. Therefore, 2 PP is suitable for manufacturing complex Three-Dimensional (3D) micron-scale or even centimeter-scale structures with nanoscale resolution [15,16,17,18,19,20]. The point-by-point scanning routine in the conventional 2 PP is inherently inefficient. Previous research has used galvanometer pairs or micro-lens arrays to greatly improve the efficiency. However, the off-axis optical aberrations and/or the limited field of view of high N.A. objectives still make the galvanometer pairs unsuitable for high-volume manufacturing. Meanwhile, the multi-focus beams generated by the static micro-lens array can perform large-area synchronous machining. However, this scheme only shows advantages in manufacturing periodic microstructures. In contrast, Spatial-Light-Modulator (SLM)-based rapid processing shows greater programmability and flexibility. To generate complex structured lights for fabricating functional microstructures, variable algorithms such as Gerchberg–Saxton, Non-Convex Optimization, and Neural Networks are widely used in customizing the corresponding Computer-Generated Holograms on SLMs [21,22,23,24,25,26]. In addition to these iterative algorithms, analytical structured beams (Bessel [27], Vortex [28], Airy [29], etc.) are also adopted in fabricating specific microstructures efficiently. With the aid of shaped beams generated by the SLM, the processing efficiency of 2 PP would be greatly improved since the time-consuming scanning for the microstructure can be simplified to a single one-step exposure. By carefully positioning the shaped beam across the interface between the substrate and the photoresist, we could acquire the desired structure rapidly with a high resolution.

As a typical case, Ring Airy Beams [30,31,32] can Abruptly Autofocus (AAF) in the linear media due to their specific initial phase and amplitude distribution, exhibiting a nonlinear shrinking of the spot size along their propagation direction. Abruptly Autofocusing Vortex (AAFV) beams are generated by imprinting the Orbital Angular Momentum (OAM) into the phases of AAF beams [33,34,35]. They appear with hollow intensity rings in the focal plane, and the size of the focal ring is determined by the topological charge. Owing to their interesting spatial geometries, AAFV beams allow sophisticated manipulations in fields including optical tweezers, optical communications, and plasma guiding.

In this paper, we demonstrate a dynamic holographic processing method to fabricate a 3D bowl-shaped micro-trap array. First, AAFV beams were generated in the direct space using a single-phase-only SLM, whose self-accelerating propagation trajectory was investigated by a set of ray-based caustics schemes. We designed two AAFV beams with different self-accelerating trajectories and inner opening diameters. The measured beam intensity profiles along the propagation showed high consistency with the numerically simulated results. On this basis, a 3D bowl-shaped micro-trap array was fabricated in the negative photoresist via the AAFV-beam-assisted 2 PP method. This strategy exhibited great tunability since the geometric features of AAFV beams can be customized in real-time by changing the phase mask loaded on the SLM. Furthermore, the intrinsic correlations between the exposure dose and the dimensions (inner opening diameter, height, and wall thickness) of the fabricated structures were revealed through the analyses of the SEM results. Finally, effective trapping of particles (polystyrene microsphere) is demonstrated using the manufactured micro-trap array, and the methods may find applications in fiber optics and cell studies.

## 2. Phase Mask Design and Experimental Setup

We used the AAF beams generated in the direct space based on our previous work [31]. These AAF beams can be synthesized from the axially symmetric self-accelerating beams along polynomial curves, which can be written as follows:

(1) $$r=r_{0}-a\times {\left(sz\right)}^{n},\;n=2,3,4,\cdots$$

where *r*_0_ is the radius of the central opaque disk, *a* is the shape factor, and *s* is the axial scaling factor (*s* ≥ 0). The AAF beam with *s* = 1 was used as the reference. Correspondingly, the geometric focus of the acceleration trajectory is located in the plane:

(2) $$Z_{c}=\frac{1}{s}{\left(\frac{r_{0}}{a}\right)}^{\frac{1}{n}}$$

Therefore, the phase mask to generate the AAF beams can be expressed as [31]:

(3) $$\phi_{AAF}=-sk\frac{n^{2}}{\left(2n-1)(n-1\right)}{\left[(n-1)a\right]}^{\frac{1}{n}}{\left(r-r_{0}\right)}^{\frac{2n-1}{n}}$$

Adding the phase of the spiral phase plate to imprint an Orbital Angular Momentum (OAM), the total phase mask of the AAFV beams can be written as [31]:

(4) $$\phi (r,\theta )=\phi_{AAF}+\phi_{vortex}=-sk\frac{n^{2}}{\left(2n-1)(n-1\right)}{\left[(n-1)a\right]}^{\frac{1}{n}}{\left(r-r_{0}\right)}^{\frac{2n-1}{n}}+m\theta$$

where *k* is the wave number and *m* is the topological charge of the optical vortex. We noticed that the main geometric features of the AAFV beams, such as the initial spot radius, focal position, and spot radius at the focal plane can be flexibly modulated by the parameters *r*_0_, *a*, and *m*. Under the joint manipulation of the above parameters, the generated AAFV beam had highly tunable geometric features, paving the way for real-time dynamic switching of phase masks during the manufacturing process.

**Figure 1:**
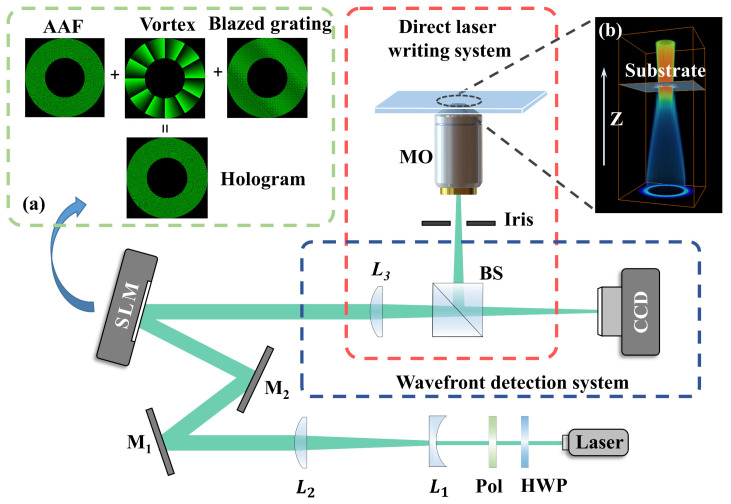
The schematic of the experimental setup. (**a**) Hologram loaded on the spatial light modulator. (**b**) Schematic of the relative position of the AAFV beams and the negative photoresist spin-coated on a glass substrate. HWP: Half-Wave Plate; Pol: Polarizer; M_1_ and M_2_: Mirrors; SLM: Spatial Light Modulator; L_1_, L_2_, and L_3_: Lens; BS: Beam Splitter; MO: Microscope Objective, CCD: CCD camera.

The experimental setup for the 2 PP process is shown in Figure 1. The laser source was a home-made femtosecond fiber laser amplification system, enabling output laser pulses at a 1 MHz repetition rate [36]. Through the Second-Harmonic Generation in a BBO crystal, a 525 nm femtosecond laser was obtained to initiate two-photon absorption. Expanded by the Galileo-type telescope system consisting of two lenses L_1_ and L_2_ (focal length *f*_1_ = −50 mm, *f*_2_ = 200 mm), the incident beam on the SLM (Holoeye PLUTO, Volmerstrasse 1, 12489 Berlin, Germany, 1920 × 1080 pixels) was transformed into the AAFV beam. As shown in Figure 1a, the phase mask loaded on the SLM consisted of three parts, the phase of AAF beams, the optical vortex, and a blazed grating, respectively. Among them, the superposition of a blazed grating directs the field of the shaped beam to the −1st order, so the undesired diffraction orders could be filtered out by the iris mounted close to the objective (MO). Besides, the hollow intensity ring due to the introduction of optical vortex resulted in an increased contact area between the polymer and the substrate in the case of AAFV beams, making the structure more robust and less likely to fall off from the substrate. The lens L_3_ (focal length *f*_3_ = 1000 mm) and the Microscope Objective (MO) (Olympus 50×, NA = 0.8) formed a relay optical system to shrink the AAFV beams, which guaranteed a higher energy density and direct writing resolution. The wave-front-detection system consisting of L_3_ and a CCD located in its focal plane realized the visualization of modulated beams and the optical aberration compensation.

## 3. Numerical and Experimental Results

Taking two sets of parameters of the AAFV beams as examples, by loading the corresponding phase masks on the SLM, we generated AAFV beams with two different accelerating trajectories in the direct space. Meanwhile, to compensate for the optical aberrations mainly caused by the spatial walk-off effect in the SHG, we used the method proposed in [37,38] to realize in situ aberrations’ correction, thus improving the optical quality of the modulated AAFV beams and the quality of the fabricated structures.

**Figure 2:**
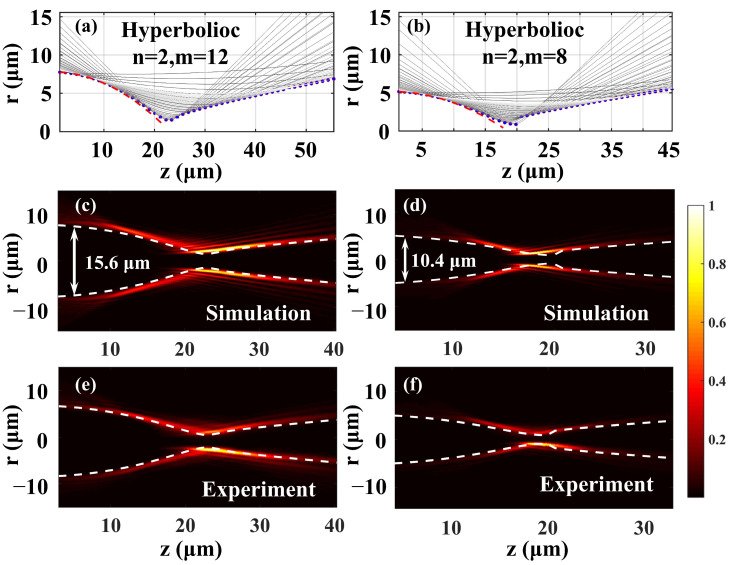
The numerical simulations and experimental results of AAFV beams with different trajectory parameters; left column: *n* = 2, *m* = 12, *a* = 0.68 × 10^−6^ mm^−1^, *r*_0_ =2.16 mm; right column: *n* = 2, *m* = 8, *a* = 0.7 × 10^−6^ mm^−1^, *r*_0_ =1.44 mm. (**a**,**b**) Hyperboloid projection of AAFV beams with two trajectories (gray solid line) and global caustic (blue dots); the trajectory of the corresponding AAF beams is marked with the red dotted line. (**c**,**d**) Numerical simulations. (**e**,**f**) Experimental results. The white dashed lines are the pre-designed caustics’ trajectory of AAFV beams.

The caustics, numerical simulations, and experimental results are shown in Figure 2. The global caustic can be calculated with the ray-based methods from our previous work [34], and the corresponding parameters in Equation (3) were *n* = 2, *m* = 12, *a* = 0.68 × 10^−6^ mm^−1^, *r*_0_ = 2.16 mm (Trajectory A, left column in Figure 2) and Figure 2b: *n* = 2, *m* = 8, *a* = 0.7 × 10^−6^ mm^−1^, *r*_0_ = 1.44 mm (Trajectory B, right column in Figure 2). According to the conclusions in [34], the rays emerging from the ring with a given radius of r in the initial plane z = 0 formed a single hyperboloid, and the hyperboloids for different *r* would superimpose and form the global caustic. Figure 2a,b plot the projections of hyperboloids in the x-z plane (solid gray lines) formed by a cluster of rays emerging from initial rings with different radii. The blue dots in Figure 2a,b correspond to the projections of these two caustic trajectories in the x-z plane. The polynomial trajectories of the corresponding AAF beams without OAM are also plotted with red dashed lines for comparison. It is evident that the caustics of these two AAF beams with and without OAM were almost indistinguishable before the focal region. The pre-designed caustics’ trajectories (white dashed lines) also agreed well with the longitudinal intensity profiles of the AAFV beams in Figure 2c–f.

Figure 2c,d numerically depict the propagating features of the AAFV beams. The lateral accelerating caustic of the AAFV beams is evident, indicating that the energies in the main ring concentrate along propagation in an accelerated manner. Close to the focal plane, the power of the AAFV beams was confined in a small ring. After focusing, the ring intensity began to attenuate. All the experimental results (Figure 2e,f) showed excellent agreement with the theoretical results.

**Figure 3:**
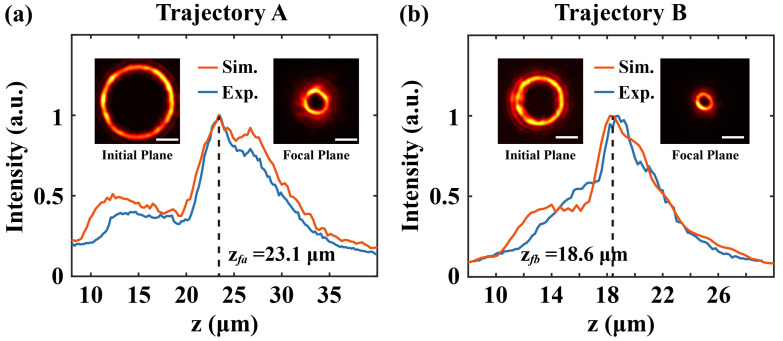
The peak intensity evolutions of the AAFV beams along propagation: numerical (orange solid lines) and experimental (blue solid lines) results. (**a**) Trajectory A. (**b**) Trajectory B. The inset shows the beams’ intensity profiles in the initial transverse plane and in the focal plane (scaling bars without notation represent 5 μm).

Figure 3 shows the peak intensity profiles of the ring in the AAFV beams along propagation. Considering the scaling factor of the relay optical system, the initial diameters of the AAFV beams in the focal plane of the microscope objective were 15.6 μm and 10.4 μm, and the theoretical axial focal positions [34] were *Z_fA_* = 23.1 μm and *Z_fB_* = 18.6 μm, respectively. The measured beam intensity profiles in the initial plane and in the focal plane are also given in the inset of Figure 3. Thanks to the lateral acceleration, the diameter of the rings rapidly collapsed to 4.4 μm (*m* = 12) and 2.6 μm (*m* = 8) along the propagation. It is noteworthy that the beam profiles at these two locations exhibited a certain degree of intensity non-uniformity, and the reasons for this beam quality degradation were various. On the one hand, the limited aperture of the setup (e.g., the pupil of the microscope objective) inherently served as a low-pass filter in the spatial spectra of the AAFV beams, filtering the high-frequency information. Therefore, the physical limits of the setup should be carefully considered when designing the self-accelerating trajectory. Another non-negligible factor originated within the optical system, such as residual optical aberrations, assembly errors of the relay optics system, the slight lateral misalignment between the center of the phase mask and the peak intensity of the incident Gaussian beams, etc. However, the resulting deficiencies in beam quality did not adversely affect the pre-designed geometric trajectory and was generally acceptable.

In the 2 PP experiment, the sample was prepared by spin-coating the negative photoresist (MicroChem SU-8 2075, 200 Flanders Road, Westborough, MA 01581, USA, diluted with acetone, with a ratio of SU-8: acetone = 5:1) onto standard slide substrates at a speed of 2100 rpm. Soft baking was accomplished on a hot plate for 60 min at 65 °C to evaporate the solvent in the photoresist. Here, we selected the structured beams before the focal plane for two-photon polymerization, whose shape was similar to a bowl. A concern may arise from the occurrence of vortex decay observed for high-order optical vortices [39]. The beam profiles in Figure 3 confirm that the ring features were maintained without vortex splitting through the whole beam volume without visible decay in the linear regime. The noise level induced by nonlinearity was proven to have an insignificant effect on this issue [39,40]. During the exposure, the focus of the AAFV beams needed to be precisely positioned across the interface between the photoresist and the glass substrate. A schematic of the relative position of the AAFV beams and the negative photoresist is shown in Figure 1b. Since the exposing dose of the volume in front of the focus exceeded the polymerization threshold of the photoresist, a single one-step exposure was sufficient for fabricating each bowl-shaped microstructure. In other words, during the whole exposure process, the spatial 3D structure forming did not need the movement of the z-axis translating stage, and this advantage will significantly reduce the manufacturing time. After post-baking at 65 °C and being developed in acetone, the polymerized structures could be retained.

**Figure 4:**
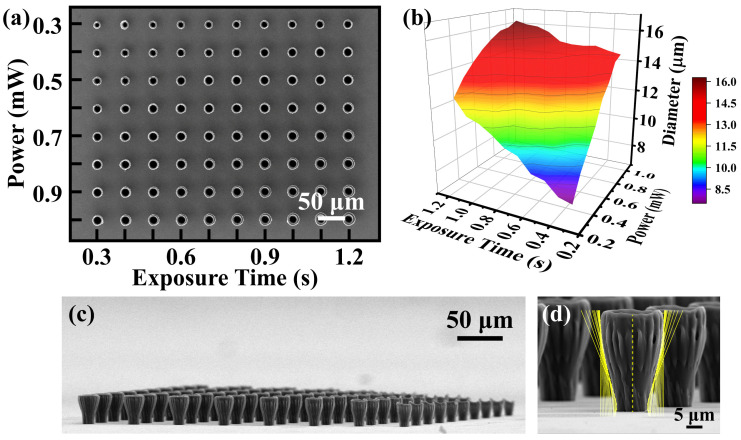
Abruptly autofocusing vortex beams enable two-photon polymerization: (**a**) For the exposure parameters, the laser power ranges from 0.3 mW to 1.0 mW with steps of 0.1 mW and the exposure times from 0.3 s to 1.2 s with steps of 0.1 s. (**b**) Calculated inner opening diameter at different laser power and exposure times. (**c**) SEM image of fabricated structures. (**d**) Comparison of structural contours with pre-designed self-accelerating trajectory.

Before the formal manufacture of micro-traps, it was necessary to analyze the relationship between the geometric size of the microstructure and the exposure dose quantitatively. In this section, an incremental exposure dose was adopted with the power and exposure time as variables. Eight laser average power steps from 0.3 mW to 1.0 mW and ten exposure time steps from 0.3 s to 1.2 s were selected, respectively. The self-accelerating parameters in Trajectory A were adopted. The SEM results of the incremental exposed micro-traps are shown in Figure 4 (TESCAN MIRA LMS). Figure 4a shows the SEM image (top view) of the bowl-shaped polymer array fabricated under different exposure doses, and Figure 4b shows the evolution of the inner opening diameter of the polymer as the power and exposure time changed. As the exposure dose increased, the inner opening diameter of the fabricated microstructures increased rapidly from the initial 7.5 μm. Until the laser power was higher than 0.8 mW and the exposure time was longer than 0.9 s, the diameter gradually stabilized and approached 16 μm, indicating that the exposure dose tended to saturate. The exposure dose in the upper left region of Figure 4a was insufficient such that only the region near the focal point could initialize two-photon absorption. We can observe the growing opening diameters of the microstructure with the increase of the exposure dose and axially increased self-accelerating trajectories.

Besides the opening diameter, the underexposure state also resulted in the reduced height of the polymer, as shown in the SEM photos in Figure 4c. This intuitively demonstrated that the significantly polymerized regions enlarged with the increased exposure doses. When the exposure parameters were 0.3 mW and 0.3 s, the height of the microstructure was about 14.6 μm, much lower than the pre-designed *Z_fA_* = 23.1 μm. By increasing the exposure dose, the visibly polymerized position moved axially toward the initial plane, and the height of the microstructure gradually approached 25 μm, matching the pre-designed trajectory. Figure 4d illustrates the comparison between the microstructure (exposure doses of 0.5 mw and 1.2 s) and the pre-designed Trajectory A, exhibiting excellent consistency. It is obvious that the outer surface profile of the polymer inherited the AAFV beams’ self-accelerating properties.

**Figure 5:**
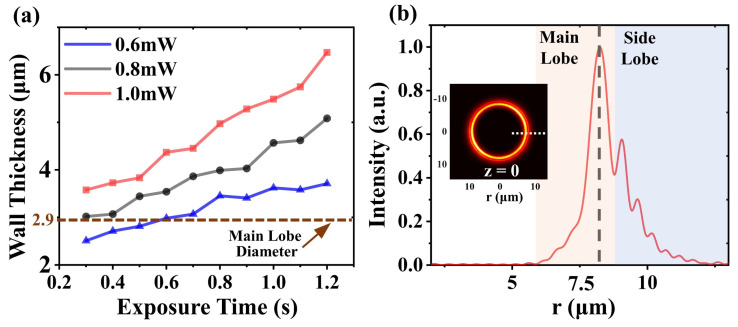
(**a**) The wall thickness evolution curves with increasing exposure dose. The brown dashed line marks the main-lobe diameter of the AAFV beams at the initial propagation position (*z* = 0). (**b**) Intensity profile at the initial position of the AAFV beam (*z* = 0) (plotted along the white dashed line in the subplot).

The wall thickness of the microstructure was influenced not only by the beam uniformity, but also the exposure dose. We plot the variation curves of the wall thickness at three laser average powers in Figure 5a. The wall thickness exhibited a significant thickening trend with the increase of the exposure time (exposure dose). Compared to the theoretically calculated main-lobe diameter (brown dotted line in Figure 5a), the experimentally measured wall thicknesses were significantly larger, which could be attributed to the side-lobe (blue-gray region in Figure 5b) with high intensities above the 2 PP threshold.

**Figure 6:**
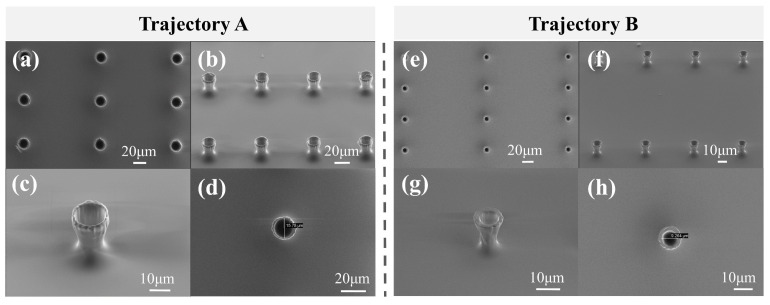
The SEM results of the bowl-shaped micro-trap arrays fabricated by the AAFV beams with Trajectory A (left column) and Trajectory B (right column). (**a**–**d**) Fabricated by the AAFV beams with Trajectory A. (**e**–**h**) Fabricated by the AAFV beams with Trajectory B.

The exposure parameters used for the formal fabrication of the micro-trap array were an average power of 0.5 mW (with a single-pulse energy of 0.5 nJ) and an exposure time of only 0.8 s. Considering the time-consuming position switching by the X-Y stage (MLS203, Thorlabs, Newton, New Jersey, United States), the micro-trap array (10 × 10) was fabricated within 3 min. The SEM images are shown in Figure 6. The two types of micro-traps fabricated by the AAFV beams with the self-accelerating Trajectories A and B are shown in Figure 6a–h, respectively. Furthermore, the fabrication of the micro-traps with other opening diameters only required dynamically switching the phase mask loaded on the SLM. The measured inner opening diameters of these two types of micro-trap were 15.8 μm and 9.3 μm, respectively. Table 1 summarizes the comparison of the key parameters between the two types of micro-traps.

## 4. Three-Dimensional Bowl-Shaped Micro-Traps for Trapping Microspheres

**Figure 7:**
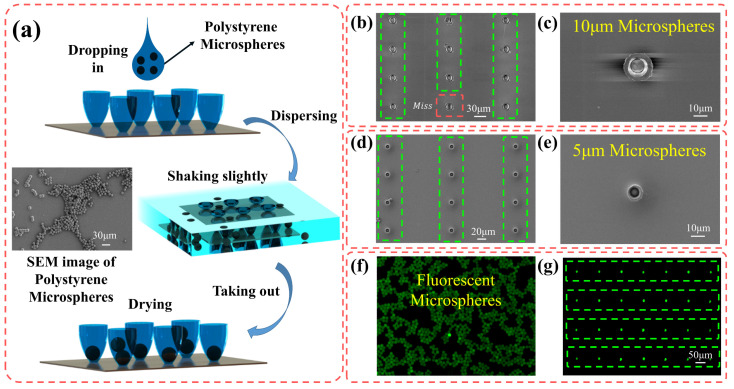
Bowl-shaped micro-trap array for trapping microspheres, the green dashed boxes indicate the micro-traps capturing the single microsphere successfully, while the red one indicate missing. (**a**) Procedures for the trapping of polystyrene particles. (**b**–**e**) The SEM images (top view) of the capture results utilizing micro-traps with different opening diameters. (**b**,**c**) An opening diameter of 10 μm. (**d**,**e**) An opening diameter of 5 μm. (**f**,**g**) The fluorescence images (top view) of the polystyrene microspheres before and after being trapped by the micro-trap array.

The ability to trap and position micro-objects shows high potential in microfluidic chip and cell growth kinetics research, especially in studying cellular heterogeneity at the single-cell level. In this part, we demonstrate the feasibility of trapping particles using the 3D micro-trap array fabricated via the AAFV-beam-assisted 2 PP method, as illustrated in Figure 7a. The sample was prepared by dropping the absolute ethanol solution containing mono-disperse polystyrene microspheres on the glass substrate, where the diameter of the polystyrene microsphere was slightly smaller than the opening diameter of the micro-traps. As the solvent evaporated, a part of the microspheres was trapped in the bowl-shaped micro-trap array under the combined effects of gravity and hydrodynamics. Next, the sample was washed alternately with dehydrated ethanol and deionized water and was gently shaken to flush the non-trapped microspheres away. After drying, the trapped microspheres could be trapped in the bowl-shaped polymer. Except for increasing the concentration of microspheres in the solvent, repeating the above procedures could also improve the success rate of capturing. Once a single-trap structure in the array had successfully captured a microsphere, the trap was “blocked”, preventing the entry of other microspheres.

The customizable bowl-shaped micro-traps can be used to capture microspheres with different diameters. Two micro-trap arrays with different opening diameters were manufactured through our dynamic holographic processing method using the AAFV beams with pre-designed self-accelerating Trajectories A and B, respectively. On this basis, the capture of polystyrene microspheres with diameters of 10 μm and 5 μm could be accomplished. The SEM results (top view) are shown in Figure 7b–e. The capture efficiency of the micro-traps is a key factor to evaluate its trapping capability, and here, we defined the capture efficiency as the ratio of the number of successfully captured polystyrene microspheres to the total number of micro-traps. The micro-traps marked by the green dashed box indicated that the target single microsphere was captured, while the red one indicated missing, and the overall success rate of capturing was above 90% (the microsphere capture process was carried out only once). To demonstrate the functionality of the micro-trap arrays more intuitively, re-arranging the photoluminescent microspheres was realized with our micro-traps. In the fluorescence images (Figure 7f,g) obtained by the confocal microscope (Olympus FV1000/IX81, Shinjuku Monolith, 3-1 Nishi-Shinjuku 2-chome, Shinjuku-ku, Tokyo 163-0914, Japan, inverted), the microspheres with a diameter of 10 μm emitted green fluorescence with a maximum wavelength of 518 nm under the excitation of a 473 nm laser. Initially, the fluorescent microspheres were randomly scattered in the Petri dishes (Figure 7f), but after capturing, these microspheres were well-positioned in a predesigned pattern (Figure 7g). Theoretically, to capture microspheres of other sizes, we only need to redesign the self-acceleration trajectory and load the corresponding phase mask on the SLM to re-fabricate the micro-trap arrays through our tunable AAFV-beam-assisted 2 PP method.

## 5. Conclusions

In this paper, we successfully fabricated a simple and tunable 3D microsphere capture device based on the dynamic holographic 2 PP method in which a single-phase-only SLM was the core element. Two kinds of AAFV beams with different self-accelerating trajectories were pre-designed by means of caustic analysis, and their spatial propagation characteristics were theoretically and experimentally investigated. Moreover, bowl-shaped micro-trap arrays with different opening diameters could be fabricated through the AAFV-beam-assisted one-step exposure strategy. By controlling the exposure dose, the relationship between the exposure dose and the geometric features of the fabricated microstructures were investigated through the analyses of the SEM images.

We demonstrated that precise positioning of a single polystyrene microsphere could be realized with this device, as the microspheres with a diameter of 10 μm or 5 μm were captured experimentally. We believe these customizable 3D-microsphere-capture devices acting as a capture–hold–analyze system can be very useful in broad applications, such as fiber optics, biological research, and fluorescent materials.

## Figures and Tables

**Table 1:** Comparison of the two types of micro-traps.

Parameters	Type 1	Type 2
Self-Accelerating Trajectory	Trajectory A	Trajectory B
Inner Opening Diameters	15.8 μm	9.3 μm
Design Height	23.1 μm	18.6 μm
Target Microsphere Diameter	10 μm	5 μm

## Data Availability

The data presented in this study are available upon request from the corresponding author.

## References

[1] Martella D., Nocentini S., Nuzhdin D., Parmeggiani C., Wiersma D.S. (2017). Photonic Microhand with Autonomous Action. Adv. Mater..

[2] Rus D., Tolley M.T. (2015). Design, Fabrication and Control of Soft Robots. Nature.

[3] Lindström S., Andersson-Svahn H. (2010). Overview of single-cell analyses: Microdevices and applications. Lab Chip.

[4] Matuła K., Rivello F., Huck W.T.S. (2020). Single-Cell Analysis Using Droplet Microfluidics. Adv. Biosyst..

[5] Omori R., Kobayashi T., Suzuki A. (1997). Observation of a Single-Beam Gradient-Force Optical Trap for Dielectric Particles in Air. Opt. Lett..

[6] Zhang H., Liu K.-K. (2008). Optical Tweezers for Single Cells. J. R. Soc. Interface.

[7] Yu J., Xu Y., Lin S., Zhu X., Gbur G., Cai Y. (2022). Longitudinal Optical Trapping and Manipulating Rayleigh Particles by Spatial Nonuniform Coherence Engineering. Phys. Rev. A.

[8] Zhang Y., Min C., Dou X., Wang X., Urbach H.P., Somekh M.G., Yuan X. (2021). Plasmonic Tweezers: For Nanoscale Optical Trapping and Beyond. Light Sci. Appl..

[9] Killian J.L., Ye F., Wang M.D. (2018). Optical Tweezers: A Force to Be Reckoned With. Cell.

[10] Li X., Zhou Y., Cai Y., Zhang Y., Yan S., Li M., Li R., Yao B. (2021). Generation of Hybrid Optical Trap Array by Holographic Optical Tweezers. Front. Phys..

[11] Pokroy B., Kang S.H., Mahadevan L., Aizenberg J. (2009). Self-Organization of a Mesoscale Bristle into Ordered, Hierarchical Helical Assemblies. Science.

[12] Jo M.C., Liu W., Gu L., Dang W., Qin L. (2015). High-Throughput Analysis of Yeast Replicative Aging Using a Microfluidic System. Proc. Natl. Acad. Sci. USA.

[13] Chen K.L., Crane M.M., Kaeberlein M. (2017). Microfluidic Technologies for Yeast Replicative Lifespan Studies. Mech. Ageing Dev..

[14] Malachowski K., Jamal M., Jin Q., Polat B., Morris C.J., Gracias D.H. (2014). Self-Folding Single Cell Grippers. Nano Lett..

[15] Kawata S., Sun H.B., Tanaka T., Takada K. (2001). Finer features for functional microdevices. Nature.

[16] Chu W., Tan Y., Wang P., Xu J., Li W., Qi J., Cheng Y. (2018). Centimeter-Height 3D Printing with Femtosecond Laser Two-Photon Polymerization. Adv. Mater. Technol..

[17] Frenzel T., Kadic M., Wegener M. (2017). Three-Dimensional Mechanical Metamaterials with a Twist. Science.

[18] Im J., Liu Y., Hu Q., Trindade G.F., Parmenter C., Fay M., He Y., Irvine D.J., Tuck C., Wildman R.D. (2023). Strategies for Integrating Metal Nanoparticles with Two-Photon Polymerization Process: Toward High Resolution Functional Additive Manufacturing. Adv. Funct. Mater..

[19] del Pozo M., Delaney C., Pilz da Cunha M., Debije M.G., Florea L., Schenning A.P.H.J. (2022). Temperature-Responsive 4D Liquid Crystal Microactuators Fabricated by Direct Laser Writing by Two-Photon Polymerization. Small Struct..

[20] Fiedor P., Ortyl J. (2020). A New Approach to Micromachining: High-Precision and Innovative Additive Manufacturing Solutions Based on Photopolymerization Technology. Materials.

[21] Wang C., Yang L., Hu Y., Rao S., Wang Y., Pan D., Ji S., Zhang C., Su Y., Zhu W. (2019). Femtosecond Mathieu Beams for Rapid Controllable Fabrication of Complex Microcages and Application in Trapping Microobjects. ACS Nano.

[22] Salter P.S., Booth M.J. (2019). Adaptive Optics in Laser Processing. Light Sci. Appl..

[23] Cheng H., Golvari P., Xia C., Sun M., Zhang M., Kuebler S.M., Yu X. (2022). High-Throughput Microfabrication of Axially Tunable Helices. Photonics Res..

[24] Manousidaki M., Papazoglou D.G., Farsari M., Tzortzakis S. (2016). Abruptly Autofocusing Beams Enable Advanced Multiscale Photo-Polymerization. Optica.

[25] Wen J., Sun Q., Luo M., Ma C., Yang Z., Su C., Cao C., Zhu D., Ding C., Xu L. (2022). Fabrication of Chiral 3D Microstructure Using Tightly Focused Multiramp Helico-Conical Optical Beams. Micromachines.

[26] Zhang J., Pégard N., Zhong J., Adesnik H., Waller L. (2017). 3D Computer-Generated Holography by Non-Convex Optimization. Optica.

[27] Khonina S.N., Kazanskiy N.L., Karpeev S.V., Butt M.A. (2020). Bessel Beam: Significance and Applications—A Progressive Review. Micromachines.

[28] Zhang H., Zeng J., Lu X., Wang Z., Zhao C., Cai Y. (2022). Review on Fractional Vortex Beam. Nanophotonics.

[29] Efremidis N.K., Chen Z., Segev M., Christodoulides D.N. (2019). Airy Beams and Accelerating Waves: An Overview of Recent Advances. Optica.

[30] Chremmos I., Efremidis N.K., Christodoulides D.N. (2011). Pre-Engineered Abruptly Autofocusing Beams. Opt. Lett..

[31] Zhao Z., Xie C., Ni D., Zhang Y., Li Y., Courvoisier F., Hu M. (2017). Scaling the Abruptly Autofocusing Beams in the Direct-Space. Opt. Express.

[32] Goutsoulas M., Efremidis N.K. (2018). Precise Amplitude, Trajectory, and Beam-Width Control of Accelerating and Abruptly Autofocusing Beams. Phys. Rev. A.

[33] Davis J.A., Cottrell D., Sand D. (2012). Abruptly Autofocusing Vortex Beams. Opt. Express.

[34] Xiao N., Xie C., Jia E., Li J., Giust R., Courvoisier F., Hu M. (2021). Caustic Interpretation of the Abruptly Autofocusing Vortex Beams. Opt. Express.

[35] Papazoglou D.G., Efremidis N.K., Christodoulides D.N., Tzortzakis S. (2011). Observation of Abruptly Autofocusing Waves. Opt. Lett..

[36] Song H., Liu B., Li Y., Song Y., He H., Chai L., Hu M., Wang C. (2017). Practical 24-Fs, 1-ΜJ, 1-MHz Yb-Fiber Laser Amplification System. Opt. Express.

[37] Čižmár T., Mazilu M., Dholakia K. (2010). In Situ Wavefront Correction and Its Application to Micromanipulation. Nat. Photon.

[38] Jia E., Xie C., Xiao N., Courvoisier F., Hu M. (2023). Two-Photon Polymerization of Femtosecond High-Order Bessel Beams with Aberration Correction. Chin. Opt. Lett..

[39] Mamaev A.V., Saffman M., Zozulya A.A. (1997). Decay of high order optical vortices in anisotropic nonlinear optical media. Phys. Rev. Lett..

[40] Xie C., Jukna V., Milian C., Giust R., Ouadghiri-Idrissi I., Itina T., Dudley J.M., Couairon A., Courvoisier F. (2015). Tubular filamentation for laser material processing. Sci. Rep..

